# Dibromido(4,4′-dimethyl-2,2′-bipyridine-κ^2^ *N*,*N*′)zinc(II)

**DOI:** 10.1107/S1600536810028692

**Published:** 2010-07-24

**Authors:** Robabeh Alizadeh, Parisa Mohammadi Eshlaghi, Vahid Amani

**Affiliations:** a School of Chemistry, Damghan University, Damghan, Iran; b Islamic Azad University, Shahr-e-Rey Branch, Tehran, Iran

## Abstract

The asymmetric unit of the title compound, [ZnBr_2_(C_12_H_12_N_2_)], contains two half-mol­ecules; both are completed by crystallographic twofold axes running through the Zn^II^ atoms which are coordinated by an *N*,*N*′-bidentate 4,4′-dimethyl-2,2′-bipyridine ligand and two Br^−^ ions, resulting in distorted ZnN_2_Br_2_ tetra­hedral coordination geometries. In the crystal, C—H⋯Br inter­actions link the mol­ecules.

## Related literature

For related structures, see: Ahmadi *et al.* (2008 ▶); Amani *et al.* (2009 ▶); Bellusci *et al.* (2008 ▶); Hojjat Kashani *et al.* (2008 ▶); Kalateh *et al.* (2008 ▶, 2010 ▶); Sakamoto *et al.* (2004 ▶); Sofetis *et al.* (2006 ▶); Willett *et al.* (2001 ▶); Yoshikawa *et al.* (2003 ▶); Yousefi *et al.* (2008 ▶).
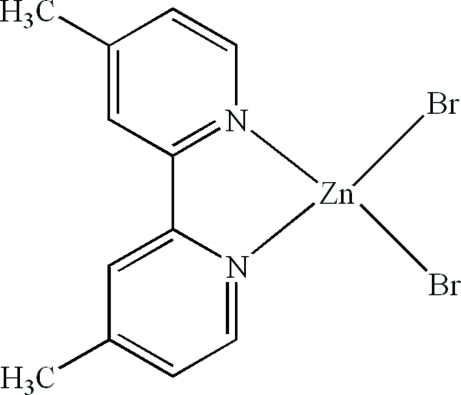


## Experimental

### 

#### Crystal data

[ZnBr_2_(C_12_H_12_N_2_)]
*M* *_r_* = 409.43
Monoclinic, 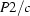

*a* = 13.801 (3) Å
*b* = 8.2454 (16) Å
*c* = 13.716 (3) Å
β = 117.47 (3)°
*V* = 1384.9 (6) Å^3^
*Z* = 4
Mo *K*α radiation
μ = 7.52 mm^−1^
*T* = 120 K
0.30 × 0.22 × 0.10 mm

#### Data collection

Bruker SMART CCD diffractometer
Absorption correction: multi-scan (*SADABS*; Bruker, 1998 ▶) *T*_min_ = 0.157, *T*_max_ = 0.479
8352 measured reflections
3560 independent reflections
2963 reflections with *I* > 2σ(*I*)
*R*_int_ = 0.050

#### Refinement

*R*[*F* ^2^ > 2σ(*F* ^2^)] = 0.039
*wR*(*F* ^2^) = 0.099
*S* = 1.07
3560 reflections
156 parameters
H-atom parameters constrained
Δρ_max_ = 0.62 e Å^−3^
Δρ_min_ = −1.04 e Å^−3^

### 

Data collection: *SMART* (Bruker, 1998 ▶); cell refinement: *SAINT* (Bruker, 1998 ▶); data reduction: *SAINT*; program(s) used to solve structure: *SHELXTL* (Sheldrick, 2008 ▶); program(s) used to refine structure: *SHELXTL*; molecular graphics: *ORTEP-3* (Farrugia, 1997 ▶); software used to prepare material for publication: *WinGX* (Farrugia, 1999 ▶).

## Supplementary Material

Crystal structure: contains datablocks I, global. DOI: 10.1107/S1600536810028692/hb5556sup1.cif

Structure factors: contains datablocks I. DOI: 10.1107/S1600536810028692/hb5556Isup2.hkl

Additional supplementary materials:  crystallographic information; 3D view; checkCIF report

## Figures and Tables

**Table:** 

Zn1—N1	2.053 (3)
Zn2—N2	2.050 (3)
Zn1—Br1	2.3428 (6)
Zn2—Br2	2.3356 (9)

**Table:** 

N1—Zn1—N1^i^	80.61 (17)
N2—Zn2—N2^ii^	81.15 (17)

Symmetry codes: (i) 
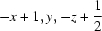
; (ii) 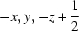.

**Table 2:** Hydrogen-bond geometry (Å, °)

*D*—H⋯*A*	*D*—H	H⋯*A*	*D*⋯*A*	*D*—H⋯*A*
C10—H10*A*⋯Br1^iii^	0.96	2.89	3.772 (5)	152

Symmetry code: (iii) 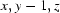.

**Fig. 1.**
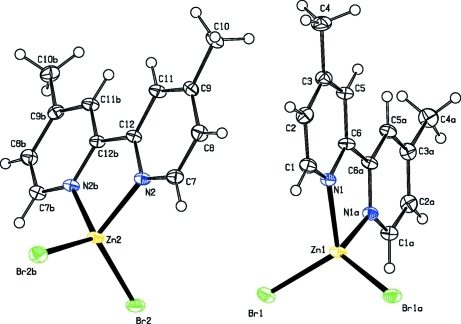
The molecular structure of (I) with displacement ellipsoids drawn at the 50% probability level. Atoms with suffix a are generated by (1–x, y, 1/2–z); those with suffix b are generated by (–x, y, 1/2–z).

**Fig. 2.**
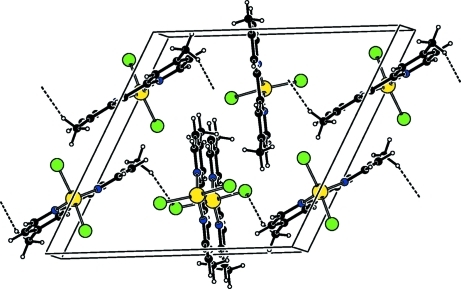
Unit-cell packing diagram for (I). Hydrogen bonds are shown as dashed lines.

## supplementary crystallographic information

### Comment

4,4'-Dimethyl-2,2'-bipyridine (4,4'-dmbipy), is a good bidentate ligand, and numerous complexes with 4,4'-dmbipy have been prepared, such as that of mercury (Kalateh *et al.*, 2008; Yousefi *et al.*, 2008), indium (Ahmadi *et al.*, 2008), iron (Amani *et al.*, 2009), platin (Hojjat Kashani *et al.*, 2008), manganese (Sakamoto *et al.*, 2004), silver (Bellusci *et al.*, 2008), gallium (Sofetis *et al.*, 2006), copper (Willett *et al.*, 2001), iridium (Yoshikawa *et al.*, 2003) and cadmium (Kalateh *et al.*, 2010). Here, we report the synthesis and structure of the title compound.

The asymmetric unit of the title compound, (Fig. 1), contains two half-molecule. The Zn^II^ atom is for-coordinated in distorted tetragonal configurations by two N atoms from one 4,4'-dimethyl-2,2'-bipyridine and two Br atoms. The Zn—Br and Zn—N bond lengths and angles are collected in Table 1.

In the crystal structure, intermolecular C—H···Br hydrogen bonds (Table 2) may stabilize the structure (Fig. 2).

### Experimental

A solution of 4,4'-dimethyl-2,2'-bipyridine (0.20 g, 1.10 mmol) in methanol (10 ml) was added to a solution of ZnBr_2_ (0.25 g, 1.10 mmol) in methanol (5 ml) at room temperature.  Colourless prisms of (I) were obtained by methanol diffusion to a colorless solution in DMSO. Suitable crystals were isolated after one week (yield; 0.35 g, 77.7%).

### Refinement

All H atoms were positioned geometrically, with C—H = 0.93Å and constrained to ride on their parent atoms, with *U*_iso_(H) = 1.2*U*_eq_(C).

### Crystal data

**Table:**

[ZnBr_2_(C_12_H_12_N_2_)]	*F*(000) = 792
*M**_r_* = 409.43	*D*_x_ = 1.964 Mg m^−^^3^
Monoclinic, *P*2/*c*	Mo *K*α radiation, λ = 0.71073 Å
Hall symbol: -P 2yc	Cell parameters from 984 reflections
*a* = 13.801 (3) Å	θ = 2.5–29.2°
*b* = 8.2454 (16) Å	µ = 7.52 mm^−^^1^
*c* = 13.716 (3) Å	*T* = 120 K
β = 117.47 (3)°	Prism, colorless
*V* = 1384.9 (6) Å^3^	0.30 × 0.22 × 0.10 mm
*Z* = 4	

### Data collection

**Table:**

Bruker SMART CCD diffractometer	3560 independent reflections
Radiation source: fine-focus sealed tube	2963 reflections with *I* > 2σ(*I*)
graphite	*R*_int_ = 0.050
phi and ω scans	θ_max_ = 29.2°, θ_min_ = 2.5°
Absorption correction: multi-scan (*SADABS*; Bruker, 1998)	*h* = −13→18
*T*_min_ = 0.157, *T*_max_ = 0.479	*k* = −11→11
8352 measured reflections	*l* = −18→17

### Refinement

**Table:**

Refinement on *F*^2^	Primary atom site location: structure-invariant direct methods
Least-squares matrix: full	Secondary atom site location: difference Fourier map
*R*[*F*^2^ > 2σ(*F*^2^)] = 0.039	Hydrogen site location: inferred from neighbouring sites
*wR*(*F*^2^) = 0.099	H-atom parameters constrained
*S* = 1.07	*w* = 1/[σ^2^(*F*_o_^2^) + (0.0449*P*)^2^ + 2.823*P*] where *P* = (*F*_o_^2^ + 2*F*_c_^2^)/3
3560 reflections	(Δ/σ)_max_ = 0.001
156 parameters	Δρ_max_ = 0.62 e Å^−^^3^
0 restraints	Δρ_min_ = −1.04 e Å^−^^3^

### Special details

**Table:**

Geometry. All e.s.d.'s (except the e.s.d. in the dihedral angle between two l.s. planes) are estimated using the full covariance matrix. The cell e.s.d.'s are taken into account individually in the estimation of e.s.d.'s in distances, angles and torsion angles; correlations between e.s.d.'s in cell parameters are only used when they are defined by crystal symmetry. An approximate (isotropic) treatment of cell e.s.d.'s is used for estimating e.s.d.'s involving l.s. planes.
Refinement. Refinement of *F*^2^ against ALL reflections. The weighted *R*-factor *wR* and goodness of fit *S* are based on *F*^2^, conventional *R*-factors *R* are based on *F*, with *F* set to zero for negative *F*^2^. The threshold expression of *F*^2^ > σ(*F*^2^) is used only for calculating *R*-factors(gt) *etc*. and is not relevant to the choice of reflections for refinement. *R*-factors based on *F*^2^ are statistically about twice as large as those based on *F*, and *R*- factors based on ALL data will be even larger.

### Fractional atomic coordinates and isotropic or equivalent isotropic displacement parameters (Å^2^)

**Table:**

	*x*	*y*	*z*	*U*_iso_*/*U*_eq_	
C1	0.3828 (3)	0.7308 (4)	0.0326 (3)	0.0225 (7)	
H1	0.3650	0.8327	0.0001	0.027*	
C2	0.3490 (3)	0.5946 (5)	−0.0343 (3)	0.0251 (8)	
H2	0.3082	0.6056	−0.1101	0.030*	
C3	0.3767 (3)	0.4417 (4)	0.0131 (3)	0.0234 (7)	
C4	0.3428 (4)	0.2903 (5)	−0.0556 (4)	0.0313 (9)	
H4A	0.2979	0.2252	−0.0345	0.038*	
H4B	0.4065	0.2299	−0.0444	0.038*	
H4C	0.3022	0.3191	−0.1318	0.038*	
C5	0.4366 (3)	0.4324 (4)	0.1268 (3)	0.0220 (7)	
H5	0.4562	0.3319	0.1611	0.026*	
C6	0.4671 (3)	0.5733 (4)	0.1892 (3)	0.0185 (7)	
C7	0.1273 (3)	0.7510 (4)	0.1512 (3)	0.0231 (7)	
H7	0.1512	0.8521	0.1411	0.028*	
C8	0.1556 (3)	0.6153 (5)	0.1110 (3)	0.0236 (7)	
H8	0.1977	0.6260	0.0746	0.028*	
C9	0.1209 (3)	0.4623 (4)	0.1250 (3)	0.0208 (7)	
C10	0.1447 (4)	0.3130 (5)	0.0775 (4)	0.0287 (8)	
H10C	0.1371	0.3370	0.0058	0.034*	
H10B	0.0943	0.2289	0.0718	0.034*	
H10A	0.2179	0.2774	0.1244	0.034*	
C11	0.0594 (3)	0.4535 (4)	0.1817 (3)	0.0218 (7)	
H11	0.0357	0.3534	0.1938	0.026*	
C12	0.0334 (3)	0.5942 (4)	0.2202 (3)	0.0181 (7)	
N1	0.4399 (3)	0.7211 (3)	0.1415 (3)	0.0186 (6)	
N2	0.0668 (3)	0.7419 (3)	0.2042 (3)	0.0186 (6)	
Zn1	0.5000	0.91089 (6)	0.2500	0.02024 (14)	
Zn2	0.0000	0.93068 (6)	0.2500	0.01997 (14)	
Br1	0.37461 (4)	1.05907 (4)	0.28513 (4)	0.02607 (11)	
Br2	0.12396 (4)	1.07654 (4)	0.40271 (4)	0.02828 (11)	

### Atomic displacement parameters (Å^2^)

**Table:**

	*U*^11^	*U*^22^	*U*^33^	*U*^12^	*U*^13^	*U*^23^
C1	0.0274 (19)	0.0185 (15)	0.0221 (19)	0.0011 (14)	0.0119 (16)	0.0019 (13)
C2	0.0263 (19)	0.0284 (18)	0.0186 (18)	−0.0019 (15)	0.0085 (15)	−0.0024 (14)
C3	0.0261 (19)	0.0232 (16)	0.0232 (19)	−0.0021 (14)	0.0134 (16)	−0.0059 (14)
C4	0.035 (2)	0.0265 (18)	0.026 (2)	−0.0044 (17)	0.0089 (18)	−0.0115 (16)
C5	0.0271 (19)	0.0141 (14)	0.0249 (19)	−0.0016 (13)	0.0121 (16)	−0.0035 (12)
C6	0.0262 (18)	0.0125 (13)	0.0187 (18)	−0.0021 (12)	0.0118 (15)	−0.0013 (12)
C7	0.0268 (19)	0.0184 (15)	0.025 (2)	−0.0015 (14)	0.0127 (16)	0.0010 (13)
C8	0.028 (2)	0.0240 (16)	0.025 (2)	0.0003 (15)	0.0171 (17)	0.0010 (14)
C9	0.0227 (18)	0.0196 (15)	0.0191 (18)	0.0039 (13)	0.0087 (15)	−0.0007 (13)
C10	0.033 (2)	0.0238 (17)	0.033 (2)	0.0066 (16)	0.0183 (19)	−0.0023 (15)
C11	0.0289 (19)	0.0138 (14)	0.0249 (19)	0.0006 (13)	0.0142 (16)	−0.0001 (12)
C12	0.0227 (17)	0.0133 (14)	0.0192 (18)	0.0012 (12)	0.0104 (15)	0.0003 (11)
N1	0.0222 (15)	0.0147 (12)	0.0177 (15)	−0.0004 (11)	0.0082 (12)	−0.0013 (10)
N2	0.0253 (16)	0.0129 (12)	0.0175 (15)	−0.0004 (11)	0.0097 (13)	0.0004 (10)
Zn1	0.0277 (3)	0.0107 (2)	0.0231 (3)	0.000	0.0123 (3)	0.000
Zn2	0.0274 (3)	0.0106 (2)	0.0212 (3)	0.000	0.0106 (3)	0.000
Br1	0.0329 (2)	0.01709 (16)	0.0322 (2)	0.00397 (14)	0.01838 (17)	0.00109 (14)
Br2	0.0317 (2)	0.01954 (17)	0.0281 (2)	−0.00180 (14)	0.00900 (17)	−0.00723 (14)

### Geometric parameters (Å, °)

**Table:**

C1—N1	1.332 (5)	C8—H8	0.9300
C1—C2	1.388 (5)	C9—C11	1.394 (5)
C1—H1	0.9300	C9—C10	1.498 (5)
C2—C3	1.388 (5)	C10—H10C	0.9600
C2—H2	0.9300	C10—H10B	0.9600
C3—C5	1.390 (6)	C10—H10A	0.9600
C3—C4	1.503 (5)	C11—C12	1.389 (5)
C4—H4A	0.9600	C11—H11	0.9300
C4—H4B	0.9600	C12—N2	1.355 (4)
C4—H4C	0.9600	C12—C12^ii^	1.488 (7)
C5—C6	1.387 (5)	Zn1—N1	2.053 (3)
C5—H5	0.9300	Zn2—N2	2.050 (3)
C6—N1	1.351 (4)	Zn1—N1^i^	2.053 (3)
C6—C6^i^	1.487 (8)	Zn1—Br1^i^	2.3428 (6)
C7—N2	1.339 (5)	Zn1—Br1	2.3428 (6)
C7—C8	1.380 (5)	Zn2—N2^ii^	2.050 (3)
C7—H7	0.9300	Zn2—Br2	2.3356 (9)
C8—C9	1.393 (5)	Zn2—Br2^ii^	2.3356 (9)
			
N1—C1—C2	122.5 (3)	C9—C10—H10C	109.5
N1—C1—H1	118.7	C9—C10—H10B	109.5
C2—C1—H1	118.7	H10C—C10—H10B	109.5
C1—C2—C3	119.3 (4)	C9—C10—H10A	109.5
C1—C2—H2	120.4	H10C—C10—H10A	109.5
C3—C2—H2	120.4	H10B—C10—H10A	109.5
C2—C3—C5	117.9 (3)	C12—C11—C9	120.0 (3)
C2—C3—C4	121.4 (4)	C12—C11—H11	120.0
C5—C3—C4	120.6 (4)	C9—C11—H11	120.0
C3—C4—H4A	109.5	N2—C12—C11	121.5 (3)
C3—C4—H4B	109.5	N2—C12—C12^ii^	115.6 (2)
H4A—C4—H4B	109.5	C11—C12—C12^ii^	122.9 (2)
C3—C4—H4C	109.5	C1—N1—C6	119.0 (3)
H4A—C4—H4C	109.5	C1—N1—Zn1	126.8 (2)
H4B—C4—H4C	109.5	C6—N1—Zn1	114.1 (2)
C6—C5—C3	120.0 (3)	C7—N2—C12	118.8 (3)
C6—C5—H5	120.0	C7—N2—Zn2	127.2 (2)
C3—C5—H5	120.0	C12—N2—Zn2	113.5 (2)
N1—C6—C5	121.3 (4)	N1—Zn1—N1^i^	80.61 (17)
N1—C6—C6^i^	115.6 (2)	N1—Zn1—Br1^i^	109.77 (9)
C5—C6—C6^i^	123.1 (2)	N1^i^—Zn1—Br1^i^	117.20 (9)
N2—C7—C8	122.2 (3)	N1—Zn1—Br1	117.20 (9)
N2—C7—H7	118.9	N1^i^—Zn1—Br1	109.77 (9)
C8—C7—H7	118.9	Br1^i^—Zn1—Br1	117.13 (3)
C7—C8—C9	120.1 (3)	N2—Zn2—N2^ii^	81.15 (17)
C7—C8—H8	119.9	N2—Zn2—Br2	114.76 (9)
C9—C8—H8	119.9	N2^ii^—Zn2—Br2	111.31 (9)
C8—C9—C11	117.4 (3)	N2—Zn2—Br2^ii^	111.31 (9)
C8—C9—C10	121.8 (3)	N2^ii^—Zn2—Br2^ii^	114.76 (9)
C11—C9—C10	120.8 (3)	Br2—Zn2—Br2^ii^	118.01 (4)
			
N1—C1—C2—C3	−1.1 (6)	C6^i^—C6—N1—Zn1	1.4 (5)
C1—C2—C3—C5	0.8 (6)	C8—C7—N2—C12	0.9 (6)
C1—C2—C3—C4	−179.6 (4)	C8—C7—N2—Zn2	−170.8 (3)
C2—C3—C5—C6	−0.2 (6)	C11—C12—N2—C7	−0.8 (6)
C4—C3—C5—C6	−179.9 (4)	C12^ii^—C12—N2—C7	179.9 (4)
C3—C5—C6—N1	−0.2 (6)	C11—C12—N2—Zn2	172.0 (3)
C3—C5—C6—C6^i^	−178.8 (4)	C12^ii^—C12—N2—Zn2	−7.3 (5)
N2—C7—C8—C9	0.0 (6)	C1—N1—Zn1—N1^i^	−177.6 (4)
C7—C8—C9—C11	−1.0 (6)	C6—N1—Zn1—N1^i^	−0.5 (2)
C7—C8—C9—C10	176.7 (4)	C1—N1—Zn1—Br1^i^	−62.0 (3)
C8—C9—C11—C12	1.0 (6)	C6—N1—Zn1—Br1^i^	115.1 (3)
C10—C9—C11—C12	−176.7 (4)	C1—N1—Zn1—Br1	74.9 (3)
C9—C11—C12—N2	−0.2 (6)	C6—N1—Zn1—Br1	−108.0 (3)
C9—C11—C12—C12^ii^	179.1 (4)	C7—N2—Zn2—N2^ii^	174.8 (4)
C2—C1—N1—C6	0.7 (6)	C12—N2—Zn2—N2^ii^	2.71 (19)
C2—C1—N1—Zn1	177.7 (3)	C7—N2—Zn2—Br2	−75.8 (3)
C5—C6—N1—C1	−0.1 (6)	C12—N2—Zn2—Br2	112.2 (3)
C6^i^—C6—N1—C1	178.7 (4)	C7—N2—Zn2—Br2^ii^	61.5 (3)
C5—C6—N1—Zn1	−177.4 (3)	C12—N2—Zn2—Br2^ii^	−110.5 (3)

Symmetry codes: (i) −*x*+1, *y*, −*z*+1/2; (ii) −*x*, *y*, −*z*+1/2.

### Hydrogen-bond geometry (Å, °)

**Table:**

*D*—H···*A*	*D*—H	H···*A*	*D*···*A*	*D*—H···*A*
C10—H10A···Br1^iii^	0.96	2.89	3.772 (5)	152

Symmetry codes: (iii) *x*, *y*−1, *z*.
